# Prevalence and determinants of adherence to antiretroviral treatment among HIV patients on first-line regimen: a cross-sectional study in Dakar, Senegal

**DOI:** 10.11604/pamj.2019.33.95.17248

**Published:** 2019-06-10

**Authors:** Mouhamed Abdou Salam Mbengue, Serigne Omar Sarr, Aissatou Diop, Cheikh Tidiane Ndour, Bara Ndiaye, Souleymane Mboup

**Affiliations:** 1 Institut de Recherche en Santé, de Surveillance Epidémiologique et de Formations (IRESSEF), Dakar, Sénégal; 2 University of the Witwatersrand, Departement of Epidemiology and Biostatistics, School of Public Health, Faculty of Heath Sciences, Johannesburg, South Africa; 3 Department of Pharmacy, Faculty of Medicine and Pharmacy Cheikh Anta Diop University, Dakar, Senegal; 4 Department of Infectious Diseases, Faculty of Medicine and Pharmacy Cheikh Anta Diop University, Dakar, Senegal; 5 Centre de Formation et de Recherche Clinique de Fann Dakar, Sénégal

**Keywords:** HIV/AIDS, antiretroviral treatment, adherence public hospitals, Senegal

## Abstract

**Introduction:**

Successful and long-term positive impact of antiretroviral treatment requires high rates of adherence (> 90%). In Senegal, there is a lack of data regarding adherence to antiretroviral treatment and only a few studies have looked at the determinants. The aim of this study is to assess the prevalence and determinants contributing to antiretroviral (ARV) adherence among Human Immunodeficiency Virus (HIV) infected outpatients receiving care at four public hospitals in Dakar, Senegal.

**Methods:**

A cross-sectional based study was carried out among HIV-positive ART adults in Dakar, Senegal. Patients were systematically sampled during either their clinical visits or visit to collect ARV drugs from six public hospitals and data collected with a questionnaire. The study outcome was adherence to antiretroviral treatment assessed by a multiple approach method which combined three self-reported adherence tools: self-reporting, Visual Analog Scale (VAS), and the Simplified Medication Adherence Questionnaire (SMAQ). Data were entered with an Excel spreadsheet and transferred to STATA for descriptive, bivariate and multivariate analysis. All the statistical tests were done at the threshold level of 0.05.

**Results:**

A total of 150 HIV-positive patients on first line ART regimen at six public health facilities were enrolled into the study. The mean age of patients was 43.1 years with a sex ratio of 0.3. Most of the patients were prescribed Tenofovir-based regimen. Of these patients, 26.67% were found to be highly adherent. After adjusting for health-related variables, demographic and socio-economic variables, better adherence was associated with participating actively within an association of persons living with HIV (AoR=2.89; 95% CI: 1.04 - 7.99; p value 0.041) while being widowed patient was associated with lower adherence (AoR=0.17; 95% CI: 0.03 - 0.94; p value 0.043).

**Conclusion:**

Our study findings imply that adherence should be routinely assessed during medical visits. Ongoing strategies to improve adherence such as out-of-clinic group-based models or psychological support should be directed toward outpatients' clinics to assist in improving adherence and long term virologic suppression in Senegal.

## Introduction

Successful and long-term positive impact of antiretroviral therapy requires high rates of adherence. There is a clear relationship between adherence to ARV treatment, viral load suppression, acquired drug resistance and treatment failure [1, 2]. In the long term, non-adherent patients on tri-therapy are more likely to die than adherent patients on the same therapy [3, 4]. In developing countries where older first-line therapies are being used, the development and transmission of drug-resistant strains of HIV will cause the switch to costly second line antiretroviral treatment and limit the treatment options available. Successful antiretroviral treatment requires sustaining high rates of adherence and is a complex behavior which is influenced by a wide range of factors. These factors have been previously categorized into socio-demographic, condition-related, treatment-related, patient-related, and interpersonal factors [5-8]. However, there is only few studies on the prevalence and the main determinants of adherence to antiretroviral treatments in the context of routine clinical care at the public health facilities. Since 1998, Senegal has launched an ambitious antiretroviral treatment programme with a free access to antiretroviral treatment and care for all HIV infected patients [9]; in 2010, there was more than 15,000 patients on antiretroviral treatment in more than 100 health facilities across the country and currently, the coverage rate for ARV among HIV adult patients in Senegal is estimated to be at 75% [10]. Since 2012, the country has adopted the WHO/UNAIDS “90-90-90” treatment target [11]. Thus, Senegal's National AIDS program will need to initiate nearly 36,450 HIV positive patients on ART in order to attain the objective of 90-90-90 during the next following years [10] because of this increase in the number of persons initiated on ART in the upcoming years, monitoring adherence will be the key strategy for the country to attain the third goal of the “90-90-90” approach and globally for the success of the ART program across the country. In Senegal, there is a lack of study investigating adherence to antiretroviral treatment in the context of routine public health care system. Most of the studies that have assessed adherence to ARVs among infants and adult patients have been implemented alongside clinical trials and have mainly recruited patients from highly specialized research centers. Thus, few of them have assessed adherence to antiretroviral treatment in the context of routine clinical care [12-15]. The aim of this study is to assess the factors contributing to ARV adherence after initiating ARVs at six public hospitals in Dakar, Senegal.

## Methods

**Design and settings:** this is a cross-sectional study of HIV positive adult patients recruited from six public hospitals from October 2015 to January 2016. Patients were attending clinic for repeat prescriptions on first line regimen in Dakar, Senegal. Data were collected during a face-to-face structured interview during their medical visits. The study sites included : Centre de recherche pour la prise en charge clinique (CRFC-Fann Center), Centre hospitalier Roi Baudoin de Guédiawaye, Institut d'Hygiène Social (IHS), Centre de Santé de Rufisque, Centre de Santé de Diamniadio and Centre de Sante de Ouakam. In these settings, the clinical staff provides care according to Senegalese Guidelines on Antiretroviral treatment defined by the Ministry of Health and the National AIDS programme [16]. Currently, more than 15,000 patients are enrolled in care in these six (6) sites with more than 2,000 having had initiated ART. The treatment programme provides patients with access to counselling, antiretroviral treatment viral load monitoring and psychosocial support.

**Eligibility criteria:** we included HIV-positive patients who were on ART following the Senegalese antiretroviral treatment guidelines. Eligible subjects were patients ≥18 years of age who initiated standard government first-line ART regimens of Tenofovir (TDF) or Zidovudine (AZT) with lamivudine (3TC) and either Efavirenz (EFV) or Nevirapine (NVP). We excluded patients who were referred from another facility or were hospitalized at the time of the study. Thus, our study only included out-patients who initiated on ART and who came for their scheduled antiretroviral (ARV) pickup or medical visits during the time of the study. For each patient, eligibility criteria were ascertained by a clinical pharmacist at each site.

**Sample size determination:** based on a literature review of previous studies, we hypothesized that the true proportion of HIV-positive patients highly adherent to antiretroviral treatment (Po) was at most 55%. Our study wanted to identify correctly with a power of 90% (zβ = 1.28) and for two-sided test and 5% significance (zα = 1.6449) a difference of at least 10% (P1: 60%), given that we used more stringent criteria which combine three self-reported adherence methods into on single multiple adherence measurement tool. Hence, we applied the Woodward formula [17]. Thus, the total patients that should be sampled was 173 (n = 173).

**Measures and adherence assessment:** self-report questionnaire, Visual Analogue Scale (VAS) and the Simplified Medication Adherence Questionnaire (SMAQ) were used. Data on each of the three tools were collected by a final year student in Pharmacy. Each of the three methods have been validated in previous studies and extensively used in routine health care settings [18].

**Self-report questionnaire:** in the self-report questionnaire, there are four questions on which the patient responded with either “yes” or “no”. A patient who answers “no” to all four questions was recorded as highly adherent, but the one whose answer is “yes” to one of the items is recorded as moderately adherent. When a patient responds “yes” to two (2) or more questions, he or she was rated as poorly adherent [18].

**Visual analog acale (VAS):** each patient was asked to mark on a scale of measurement from 0 to 100%, his or her adherence to the medication over the past 4 weeks. The results were converted to an adherence level expressed as a proportion (%) and classified into three (3) categories of adherence. Patients with result above 95% were classified as adherent and those with a result equal to or below 95% were classified as poorly adherent to antiretroviral treatment [18].

**Simplified medication adherence questionnaire (SMAQ):** the SMAQ was used to collect information on adherence over the previous 3 months period. The SMAQ score ranged from 0 to 7 with 0 corresponding to 100% adherence. A patient was considered as positive or non-adherent when a positive response was given to one of the questions, or the patient did not take any medicine over the past weekend, or had missed taking the medicine for more than 2 days over the past 3 months [19].

**Multi-method approach:** World Health Organization (WHO) recommends a multi-approach method when measuring adherence to antiretroviral treatment [19-21]. The multi-method approach tool included self-reports combined with VAS and the SMAQ. Overall adherence assessment with the multi-method approach was rated into two (2) categories: high and low. A high level of adherence corresponds to a patient who reported “no” to all questions with self-reporting, had a VAS score “yes” 95% and who is adherent following the SMAQ method. A patient who did not meet the above-mentioned criteria was classified as poorly adherent with the multi-method approach.

**Study variables:** the outcome of interest was poor adherence to ARV treatment. We considered a multi-method approach by further categorizing overall adherence as adherent or non-adherent. A patient was categorized as adherent if they answered “no” to the all the self-report questions, reported 90% VAS or more and knew the dose, time and instructions. Where responses to self-report, VAS or pill identification were less than optimal (e.g. moderately or poorly adherent), overall adherence was categorized as non-adherent. We evaluated the relationship between adherence to antiretroviral therapy, socio-demographic and economic factors. We used the cut-off values reported in previous studies and the diagnostic prediction models found in the literature data on socio-demographic and economic factors were collected as part of the interview for each patient. The socio-demographic variables included: age, gender, nationality, religion, marital status, education respondent, tobacco use, alcohol consumption, treatment duration and ART sites. The social support and economic factors included variables such as employment, ability to pay his/her medical care, having shared the HIV status, received support from family or friend, being a member of an association of persons living with HIV, participated to educative sessions on ARVs or visited a traditional healer recently or in the past. These variables were selected following a literature review and from previous studies that assessed factors associated with adherence to ARV [6, 8, 11, 15, 22, 23]. For each variable included in our analysis, we used the cut-off values known from previous studies and models found in the literature.

**Data analysis and statistical methods:** continuous variables were described as mean and standard deviation or median and interquartile range and were compared by t-test or Wilcoxon rank sum test when appropriate. Categorical variables were described by frequencies and percentages and were analyzed by chi-square test. For multivariate analysis, we considered the outcome category “none” as baseline/reference category. We considered socio-demographic characteristics as potentials confounding variables and we used the log-likelihood ratio test to select the independent variables for the multivariate model. The selection of independent variables for the model relied on their ability to improve the general model. For each variable, the results of the multinomial logistic regression were expressed as adjusted odds ratio (AoR) with its corresponding 95% confidence interval (CI) and p-values were calculated with an alpha level of 0.05. Data was captured and entered Excel spreadsheet and transferred to STATA 14 for descriptive, bivariate analysis and multivariate regression. A p-value < 0.05 was considered statistically significant.

## Results

**Figure 1:**
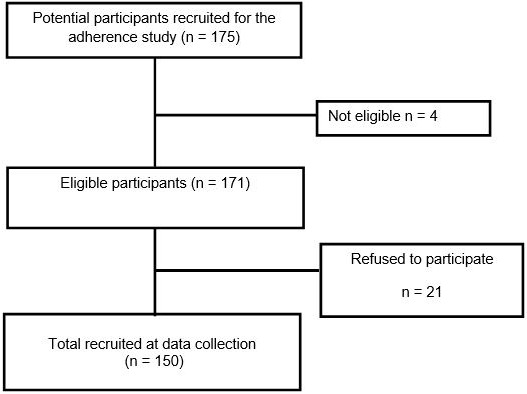
Flowchart of the participants in the cross-sectional study. The flowchart indicates how the final eligible study total participants was arrived at Participants who were pregnant, or on second line antiretroviral treatment study (n=4) were excluded as their management is different

**Sample characteristics:** 150 patients were analyzed. For the number of participants who accepted the study in detail see the flow chart below (Figure 1). Of the 150 patients in the study population, more than three quarter were females and 82.67% were aged above 35 years old. Education level ranged from beyond individuals with no education (45.33%) to secondary school (28.0%). Of the 150 patients 82.67% were Senegalese while the rest were from neighboring countries. Among the patients, only small proportions (6.67%) smoked in the past or were still smoking. 33% of patients were enrolled in CRFC-Fann Center and only 20% of patients had a treatment duration below one year. There were minimal missing values (Table 1).

**Table 1:** Demographic characteristics of HIV positive patients

Characteristics	n	% (n/N)
Age group of respondents (years)		
< 35	26	17.33
>= 35	124	82.67
**Gender**		
Man	37	24.67
Women	113	75.33
**Nationality**		
Senegalese	124	82.67
Other nationalities	26	17.33
**Religion**		
Muslim	135	90.00
Christians	15	10.00
**Marital status**		
Single	20	13.33
Divorced	23	15.33
Married	69	46.00
Widowed	38	25.33
**Education respondent**		
No education	68	45.33
Primary education	40	26.67
Secondary or higher	42	28.00
**Tobacco use**		
Yes	10	6.67
No	140	93.33
**Alcohol consumption**		
Yes	5	3.33
No	145	96.67
**Treatment duration since ART initiation**		
< 1	30	20.00
>= 1	120	80.00
**Type of HIV clinics**		
CRCF-Fann	50	33.33
Other"	100	66.67
**Employment**		
Employed	56	37.33
Unemployed	94	62.67
**Can pay his medical care**		
Yes	56	37.33
No	94	62.67
**Counseling and social support**		
**Has shared his HIV status**		
Yes	102	68.00
No	48	32.00
**Has received good social support**		
Yes	89	59.33
No	61	40/67
**Is involved into the activities of PLWVIH association**		
Yes	29	19.33
No	121	80.67
**Has once participated to education session on ARVs**		
Yes	132	88.00
No	18	12.00
**Visited a traditional healer after its diagnostic**		
Yes	8	5.33
No	142	94.67

**Social support and socio-economic characteristics:** patients were predominantly unemployed (62.67%) (Table 1). Most of the patients were prescribed TDF-3TC-EFC (81%) as first line ARV regimen. One-third (37.77%) could pay for their medical care was and 68% of the patients had shared their HIV status. Additionally, patients who participated in the activities with an association of person living with HIV (PLWHIV) were 19.33%. However, most of the patients have at least once participated to a therapeutic education session on ARVs. Finally, only 5.33% of patients have visited a traditional healer after the diagnostic of HIV infection.

**ART adherence:** a total of 150 patients had their adherence assessed using the 30-days visual analog scale (VAS) and most of them (86.7%) had a score above 95% (70.67%) in the month prior to the survey (Table 2). Results from the self-reporting adherence questionnaire, found that only 11.33% were adherent and with the SMAQ the proportion of patients classified as being highly adherent increased again to 30.6%. In the multi-method approach which combines visual analogue scale, self-reporting and SMAQ, of a total of 150 patients, 26.67% were found to be highly adherent.

**Table 2:** Adherence to antiretroviral therapy among adults

Self-Reported Adherence measures	Total	n	%
**Visual analog scale**			
VAS score > 95	105	106	70.67%
VAS score =< 95		44	29.33%
**MAQ**			
Adherent	105	46	30.67%
Non-adherent		104	69.33%
**Self-reporting adherence method**			
Adherent	105	17	11.33%
Non-adherent		133	88.67%
**Multiple method**			
Adherent	105	40	26.67%
Non-adherent		104	69.33%

**Determinants of ART adherence:** in bivariate analysis, factors associated with poor adherence were tobacco use and being involved into the activities of associations of persons living with HIV (Table 3). Results from the multivariate regression analysis (Table 4) indicates that there was a decrease in the odds of having high adherence level depending on patient's demographic and psychosocial support. Patients classified as widowed (Aor: 0.49 95% CI: 0.03-0.94. p value: 0.043) were less likely to be highly adherent as compared with patients who reported they were single at the time of the study. Additionally, patients who declared that they participate in the activities within an association of person living with VIH (PLWHIV) were 2.34 times more likely to be adherent as compared to patient who did not belong to any association of persons living with HIV (Aor:2.89 95% CI:1.04-7.99 p value: 0.041) (Table 4).

**Table 3:** Adherence to antiretroviral: Ffactors associated with therapy

	Adherence to ART			
Factors	No %	Yes %	P value	
**Age group of respondents (years)**				
< 35	19 (73.08)	7 (26.92)	0.974	
≥35	91 (73.39)	33 (26.6)		
**Gender**				
Man	29 (78.38)	8 (21.62)	0.424	
Women	81 (71.68)	32 (28.32)		
**Nationality**				
Senegalese	90 (72.58)	34(27.42)	0.761	
Others	20 (77.00)	6 (33.00)		
**Religion**				
Muslim	96 (71.11)	39 (28.89)	0.065	
Christian	14 (93.33)	1 (06.67)		
**Marital status**				
Single	13 (65.00)	7 (35.00		
Divorced	17(73.91)	6 (26.09)	0.717	
Married	50 (72.46)	19 (27.54)		
Widowed	30 (78.95)	8(21.05)		
**Respondent education**				
No education	53 (77.94)	15 (22.06)		
Primary education	27 (67.50)	13 (32.50)	0.469	
Secondary or higher	30 (71.74)	12 (28.57)		
**Tobacco use**				
Yes	10(100.00)	0 (0.00)	0.048*	
No	100 (71.43)	40 (28.57)		
**Alcohol consumption**				
Yes	4 (80.00)	1 (20.00)	0.732	
No	106 (73.10)	39 (26.90)		
**Treatment duration since ART initiation**				
< 1	22 (73.33)	8 (26.67)	1.00	
≥ 1	88 (73.33)	32 (26.67)		
**Type of HIV clinics**				
CRCF-Fann	34 (68.00)	16 (32.00)	0.296	
Other"	76 (76.00)	24 (24.00)		
**Employment**				
Employed	38 (67.86)	18 (32.14)	0.242	
Unemployed	72 (76.60)	22 (23.40)		
**Can pay his medical care?**				
Yes	49 (70.00)	21 (30.00)	0.530	
No	56 (74.67)	19 (25.33)		
**Counseling and social support**				
**Has shared his HIV status?**				
Yes	76 (74.51)	26 (25.49)	0.635	
No	34 (70.83)	14 (29.17)		
**Has received good social support?**				
Yes	66 (74.15)	23 (25.85)	0.874	
No	10 (77.00)	3 (23.00)		
**Is involved into the activities of PLWVIH association?**				
Yes	17 (58.62)	12 (41.38)	0.046*	
No	93 (76.86)	28 (23.14)		
**Has once participated to educative session on ARVs?**				
Yes	98 (74.24)	34 (25.76)	0.495	
No	12 (66.67)	06 (33.33)		
**Visited a traditional healer after its diagnostic?**				
Yes	7 (87.50)	1(12.50)	0.088	
No	103 (72.54	39 (27.46)		

**Table 4:** Determinants of adherence to antiretroviral therapy

Variables	OR	AoR	95% CI	P.value
**Age group of respondents (years)**				
< 35 (Reference)				
≥35	0.98	1.35	0.44 - 4.14	0.590
**Gender**				
Man	0.69	0.65	0.18 - 2.28	0.508
Women				
**Religion**				
Muslim	0.69	10.72	1.22- 94.36	0.032*
Christian (Reference)				
**Marital status**				
Single (Reference)				
Divorced	0.65	0.41	0.02 - 2.38	0.323
Married	0.70	0.41	1.10 -1.66	0.213
Widowed	0.49	0.17	0.03 - 0.94	0.043*
**Treatment duration since ART initiation**				
< 1 year (Reference)				
≥ 1 year	1.00	0.72	0.25- 2.1	0.544
**Type of Health Center**				
CRCF-Fann (Reference)				
Other"	0.67	0.56	0.22 -1.40	0.215
**Employment**				
Employed	1.55	1.31	0.53 - 3.25	0.552
Unemployed (Reference)				
**Can pay his own medical care?**				
Yes	1.26	1.53	0.63 - 3.73	0.341
No (Reference)				
**Is involved into the activities of PLWVIH association?**				
Yes	2.34	2.89	1.04 -7.99	0.041*
No (Reference)				
**Visited a traditional healer after its diagnostic?**				
Yes	0.37	0.539	0.20 -1.41	0.210
No (Reference)				

## Discussion

Recent estimates state that 6 million HIV-positive people have initiated ART in sub-Saharan Africa and this figure is likely to increase in the next year following the implementation of the “test and treatment initiative” and the “90-90-90” approach [16-24]. Initiating all the HIV-positive patients onto care will be a remarkable success for the National AIDS programs. However, one of the main challenges will be keeping these patients on care and highly adherent to antiretroviral treatment first line regimen. In this context, a thorough understanding of factors associated with ART adherence allows for targeted interventions that can be implemented within the context of health care services in public hospitals areas in order to keep patients in care and adhering to treatment in the early stages of ART [25]. It is therefore necessary to continue to tackle the issue of adherence and identify patients at risk of poor clinical outcomes who need therapeutic education and support. In our study, approximately 30% of patients were found to be adherent to ARV treatment. This finding is consistent with previous reports from industrialized countries, documenting 25%-44% of adherent patients [22]. However, this finding is different from previous studies in Senegal [12-15]. In a study implemented in a cohort of patients who were on ART from 1998 to 2000, Laniece *et al*. [13] showed that long term adherence among HIV patients in Dakar was very high with nearly more than 95% being adherent This situation may be explained by the fact that in our study, we used a more stringent criteria to assess adherence with the combining of three self-reported adherence as recommended by WHO [26]. Additionally, these previous studies were conducted from 1998-2000 in a highly specialised research centres with a specific cohort of HIV positive patients in the context of clinical trials with where patients had access to different services including an adherence counselling sessions and psychological support combined with regular clinical consultation. Assessing the determinants of adherence to antiretroviral treatment is critical because of its potential to target patients who need adherence counselling and support during medical visits. Our results show that patients who participated in activities with an association for persons living with HIV were more likely to achieve higher adherence level to ART compared with patients who did not participate in such activities. This finding is in line with previous studies in Africa that highlighted the positive impact and the role of peer-counselling sessions or adherence clubs for improving and maintaining high adherence levels among persons living with HIV [4, 23, 27, 28]. Our findings suggest that, participating to peer education activities and to different support groups activities could potentially help them to cope with the psychological effects of the long-term antiretroviral therapies.

Data from industrialized countries showed that younger patients with poorer education and those with low socio-economic status [29, 30] were less likely to being adherent, but there is little evidence from resource-limited settings. Surprisingly, we did not detect a relationship between adherences to antiretroviral therapies and socio-economic factors. This difference may be explained by the smaller sample size in our pilot study but also by the fact that in these previous studies, adherence was assessed in a long-term manner with a prospective cohort study. Additionally, in our study there was no association between income or socio-economic levels and adherence to ART. This situation may be explained by the fact that ART is free for all HIV positive patients regardless of their socio-economic level in Senegal since 2002 [10]. In our study, there was a significant relationship between marital status and adherence to antiretroviral treatments. Widowed patients were less likely to be highly adherent as compared to single patients. Our findings confirm previous studies in Senegal, West Africa and in Uganda who have reported a similar pattern among HIV-positive patients [15-31]. Although other studies have reported mixed results regarding the role of marital status [32, 33] on adherence, this situation may be explained by the fact that single patients may have access to better social support as they may only share their status with the medical staff for HIV antiretroviral therapy. For widowed HIV positive patients, HIV/AIDS represents a shame and they may fear discrimination and reject from the community who may already know their HIV status. Our findings should be considered within the context of the study's monitoring. First, because there is no gold standard method to assess adherence to antiretroviral treatment, thus our study used data from three self-reported adherence measures as recommended by WHO [34]. Therefore, our results may not be completely comparable to previous studies who used a single adherence questionnaire. Second, the use of self-report medication adherence was a limitation since patients had to recall the number of missed doses or the number of days during which the medication was not taken, thus there was a possibility for the participants to overestimate their adherence level. Fourth, we did not have access to ARVs medical records and informations on the availability of ARVs drugs at the health facilities. Therefore, there was no possibility to assess the impact of drug stock out in adherence levels or to correlate the adherence levels with viral load which represents the gold standard when assessing adherence to antiretroviral treatment. Finally, since this was a cross-sectional survey, the outcome and exposure were collected at the same time and therefore no inference or causalities association can be concluded. However, even with these limitations, the analysis of data collected from hospitals provides important insights into the factors influencing adherence to antiretroviral treatment in Senegal and can be useful in guiding policy implementation strategies.

## Conclusion

The adherence rate found in this study seems to be low. The use of three different adherence indicators was important for reducing bias through self-reporting and therefore strengthened the indicator. For HIV positive patients living alone particularly those who are widowed, additional supports may be needed to ensure that they obtain their medication and they comply with antiretroviral regimen. Additionally, activities such as peer to peer group-based models or adherence clubs should be implemented to assist in improving adherence and virologic suppression amongst HIV-positive patients in Senegal.

### What is known about this topic

- Adherence to an ART regimen is critical for the success of antiretroviral therapy at individual and community level;
- Many studies have shown that a low level of adherence is the most well-known reason associated with drug resistance, treatment failure and mortality in HIV-positive patients;
- However, in Senegal, there is few data regarding adherence to antiretroviral treatment among HIV positive patients in routine public health care.

### What this study adds

- Prevalence of adherence to ARV assessed with a WHO's multiple approach method;
- Determinants of adherence in urban settings and into public health facilities.

## Competing interests

The authors declare no competing interests.
